# Editorial for the Special Issue on Advanced Machine Learning Techniques for Sensing and Imaging Applications

**DOI:** 10.3390/mi13071030

**Published:** 2022-06-29

**Authors:** Bihan Wen, Zhangyang Wang

**Affiliations:** 1 School of Electrical & Electronic Engineering, Nanyang Technological University, Singapore 639798, Singapore; 2 Electrical and Computer Engineering, University of Texas at Austin, Austin, TX 78705, USA; atlaswang@utexas.edu

Recent advances in machine learning, from large-scale optimization to building deep neural networks, are increasingly being applied in the emerging field of computational sensing and imaging. A wide range of machine learning techniques, including deep learning, sparse and low-rank modeling, manifold learning, unrolled architectures, and tensor models, can be applied to enhance the effectiveness and efficiency of various sensing and imaging systems. By exploiting the underlying image or signal models via a data-driven approach, these advanced machine learning techniques benefit applications from image reconstruction to analysis.

This special issue collected and published 11 novel contributions in the imaging- and sensing-related schemes, including novel imaging and reconstruction methods, blind compressed sensing, and task-driven imaging and understanding, in which machine learning is the major component.

Advanced computational imaging techniques aim to reconstruct images with high resolution from limited measurements. Recent works showed that deep learning based super-resolution algorithms can effectively restore high-resolution images from their low-resolution measurements, which can be useful for many imaging applications. In [1], Min Zhang et al. proposed a lightweight multi-scale asymmetric attention network for single image super-resolution. The proposed deep framework includes various effective modules which are shown to be effective in exploiting the image properties via a data-driven approach. With much fewer parameters, the proposed network can achieve comparable results comparing to the most recent baselines. Furthermore, Yue Yu et al. [2] demonstrated a novel deep super-resolution scheme for Chest X-ray images that can play an important role in disease diagnosis. The key module is a wavelet frequency separation attention network, which consists of separated-path for wavelet sub-bands to predict the corresponding wavelet coefficients, assuming that the image data have their unique features in different wavelet channels. Such an effective super-resolution scheme has demonstrated competitive performance comparing to many popular lightweight medical imaging methods, by evaluating using both qualitative and quantitative metrics. Mingzheng Hou [3] proposed to apply a generative adversarial network to model extremely low-resolution images that even lack adequate scene and appearance information. Instead of only super-resolving images for visual quality, the proposed deep reconstruction scheme is coupled with a trainable analytical network, to enable reliable activity recognition from very limited imaging measurements.

Besides the resolution challenges of computational imaging, other quality degradation may also limit the downstream data analytics. In [4], Jiachao Zhang et al. proposed a novel sparse coding model, called simultaneous patch-group sparse coding (SPG-SC), which utilized dual-weighted $\ell_{p}$ in the optimization framework to exploit effective image priors. Various image reconstruction algorithms based on SPG-SC are proposed for image inpainting and deblurring, which can enhance image qualities in many imaging tasks. Furthermore, Hong Duc Nguyen et al. [5] investigated the data redundancy in X-ray imaging. Different from natural images, X-ray images that are acquired during luggage scanning and inspection usually involve small-scale objects comparing to large background. To remove the redundancy in X-ray images thus to improve the efficiency of downstream tasks, a task-driven image cropping algorithm is proposed [5] that can select only the task-relevant regions. Besdies, a novel dataset for X-ray image detection, dubbed SIXray-D, is proposed to benchmark the effectiveness of X-ray image detection algorithms. Combining the proposed task-driven X-ray image cropping scheme with deep detection algorithms, the integrated framework can further boost popular detection algorithms, with higher detection mAPs and efficiency.

Computational imaging provides reliable reconstructions of the targets, which are critical and valuable in many applications such as clinical diagnosis and remote sensing. While these images are conventionally analyzed by human, recent advances in machine learning enables automated analysis via various computer vision schemes. For clinical applications, Guanghua Xiao et al. [6] proposed a synergy factorized blinear network for brain tumor classification in magnetic resonance imaging (MRI), which is a critical task in cancer diagnosis. The proposed factorized bilinear encoding layer effectively fuses the multi-path features, with the whole network being trained in an end-to-end manner; In [7], Xujun Shu et al. proposed a three-dimensional semantic segmentation scheme for pituitary adenomas from the contrast-enhanced T1 (T1ce) images. It is achieved by training a nnU-Net model that can be used in clinical practice. Except for clinical applications, various remote sensing tasks are automated via machine learning techniques. In [8], Hongmin Gao et al. proposed a deep learning scheme for hyperspectral image classification using 3D-2D multibranch feature fusion and dense attention networks; In [9], Chenming Li et al. proposed a hybrid dilated convolution network with multi-scale residual fusion to perform a similar task for hyperspectral image classification. These deep learning based schemes can effectively improve the accuracy or robustness in hyperspectral image classification tasks over popular remote sensing benchmark hyperspectral datasets.

Except for clinic and remote sensing applications, computational imaging has also been widely used in biomedical science and industrial inspection. In [10], Shaobo Luo et al. proposed learning-based pipeline for measuring the sizes of bioparticles, which is widely used in applications such as monitoring the qualities of food and atmosphere. Different from the classic imaging-free methods, imaging acquisition using camera is capable of observing individual nanoparticles in real time. The proposed pipeline can accurately identify the cell type, followed by accurate pixel-to-size conversion for sizing tasks. In [11], Muhammad Asad Bilal Fayyaz and Christopher Johnson proposed to utilize the current visual imaging systems such as CCTVs to detect objects (e.g., pedestrians, bicycles, and vehicles) at a level crossing region in railway system. By applying deep fusion networks, the proposed system can increase the detection accuracy with lightweight models.

We would like to thank all the authors for submitting their original works to this first volume of the Micromachines Special Issue on Advanced Machine Learning Techniques for Sensing and Imaging Applications. We also appreciate the supports from all of the reviewers for dedicating their time to improve the paper quality. A special thank goes to the editor-in-chief, Prof Aiqun Liu, for his kind invitation and precious contribution, and to Mengdie Hu of the Micromachines Editorial Office for their great support.
